# Monitoring Therapy Adherence of Tuberculosis Patients by using Video-Enabled Electronic Devices 

**DOI:** 10.3201/eid2203.151620

**Published:** 2016-03

**Authors:** Alistair Story, Richard S. Garfein, Andrew Hayward, Valiantsin Rusovich, Andrei Dadu, Viorel Soltan, Alexandru Oprunenco, Kelly Collins, Rohit Sarin, Subhi Quraishi, Mukta Sharma, Giovanni Battista Migliori, Maithili Varadarajan, Dennis Falzon

**Affiliations:** Find & Treat, University College London Hospitals, London, UK (A. Story);; University of California San Diego School of Medicine, La Jolla, California, USA (R.S. Garfein, K. Collins);; Farr Institute of Health Informatics, University College London, London (A. Hayward);; World Health Organization Country Office, Minsk, Belarus (V. Rusovich);; World Health Organization (WHO) Regional Office for Europe, Copenhagen, Denmark (A. Dadu);; Center for Health Policies and Studies, Chisinau, Moldova (V. Soltan);; United Nations Development Programme, Chisinau (A. Oprunenco);; National Institute of TB & Respiratory Diseases (NITRD), New Delhi, India (R. Sarin);; ZMQ, New Delhi (S. Quraishi); WHO Country Office, Bangkok, Thailand (M. Sharma);; European Respiratory Society, Lausanne, Switzerland (G.B. Migliori);; WHO Collaborating Centre, Tradate, Italy (G.B. Migliori);; WHO Global TB Programme, Geneva, Switzerland (M. Varadarajan, D. Falzon)

**Keywords:** Tuberculosis, medication adherence, videoconferencing, telemedicine, computer communication networks, directly observed therapy, short course, DOT, video observed therapy, virtually observed therapy, VOT, individualized medicine, tuberculosis and other mycobacteria, bacteria

## Abstract

A recent innovation to help patients adhere to daily tuberculosis (TB) treatment over many months is video (or virtually) observed therapy (VOT). VOT is becoming increasingly feasible as mobile telephone applications and tablet computers become more widely available. Studies of the effectiveness of VOT in improving TB patient outcomes are being conducted.

In 2014, 1.5 million people globally died from tuberculosis (TB) (*1*). Most TB patients are eminently curable by an affordable course of treatment, although this treatment currently takes a minimum of 6 months to complete and 2 years or longer for multidrug-resistant tuberculosis (MDR-TB) (*2*). Millions of patients begin TB treatment each year but face constant challenges to comply with daily medication, causing many to adhere inconsistently or to stop prematurely. Treatment interruption increases the risk for acquired drug resistance, treatment failure, disease progression, relapse and death; it also prolongs transmissibility (*3*). Loss to medical follow-up is higher when patients have a negative treatment experience, such as when access to care involves substantial travel time, lost earnings, and other patient expenditures; when adverse drug reactions are frequent or consequential; or conversely, when patients feel better and their motivation to finish treatment declines (*4*). For many, treatment is complicated by concomitant health conditions (e.g., HIV/AIDS) and destabilizing socio-structural factors (e.g., substance abuse, homelessness, poor health care access). New medicines currently under study bring renewed hope to TB patients of safer, simpler, and more effective regimens; however, all of these treatments still require several months to complete, making adherence a continuing concern for the future (*1*).

The need for close, regular contact between caregivers and TB patients receiving treatment has been long recognized and remains topical (*5*) (http://www.who.int/tb/post2015_TBstrategy.pdf). Direct observation of treatment (directly observed therapy, or DOT) was 1 of the 5 components of the strategy promoted by the World Health Organization (WHO) and public health advocates to address the global TB emergency declared in the early 1990s (*6*), (http://www.who.int/tb/dots/whatisdots/en/). Recently, innovative approaches have been piloted that bridge the gap between caregiver and patient and limit the cost and stress of frequent travel to health centers for DOT. Telephone video communication is an example, enabling health professionals to watch patients take their medication, address patients’ concerns, and provide advice and support (*7**,**8*). Video (or virtually) observed therapy (VOT) was piloted by using videophones connected to telephone landlines and has more recently evolved toward video-enabled mobile cellular devices. Mobile telephones with video applications (smartphones) and tablet computers are becoming increasingly affordable and reliable in high- and low-income settings. Furthermore, geographic coverage of cellular and internet networks is increasingly available in places where telephone landline services had never existed or are facing obsolescence. Improved access to the technologies and infrastructure needed for VOT is foreseeable in both low- and middle-income countries in the coming years. These same countries have the greatest share of the global burden of TB and drug-resistant TB and are in urgent need of expanding their treatment programs. VOT shows promise as a new patient-centered option to support TB patients. It offers patients freedom to take their medications when and where they choose, and it engenders a more holistic approach to care.

Efforts to use VOT for TB patient support are now gaining momentum worldwide (*9*; http://www.youtube.com/watch?v=is95C8tgOyo). Studies from the United States and Mexico show that smartphone VOT is acceptable, can save resources, and improves patient commitment to treatment even in highly mobile populations (*10*). In London, United Kingdom, where DOT is recommended for treatment of patients with multidrug-resistant TB and for other patients in unfavorable social circumstances, early findings from an ongoing trial of VOT against traditional DOT is showing potential to improve adherence. Reduced costs to patients and healthcare providers are expected even when the expense of providing hardware and cellular data connection to patients are factored in (*11*). For example, a smartphone used in this study costs less than 1 episode of face-to-face contact with local community nursing. VOT has been used successfully in TB patients in London since 2007, including among children, who tend to be proficient with this technology. Another trial has started in Moldova, a middle-income former Soviet Union country in Eastern Europe, to investigate the effectiveness of VOT by using the patient’s desktop computer or a tablet computer provided by the study (*12*). A trial has also recently been launched in the United States (by R.S.G.) to compare the efficacy of VOT with traditional DOT for monitoring adherence to short-course treatment for latent TB infection. The early promise of VOT has led TB providers in Belarus, India, the United States, and elsewhere to start planning its implementation.

VOT should be viewed as a tool to facilitate patient/provider contact and not to supplant physical interaction between the patient and the healthcare professional. Patients would still need to visit clinics to collect medication, to submit samples to the laboratory, and for assessment of response to treatment. Establishing VOT also requires sound investment, including the training of patients and VOT observers. 

VOT remains a relatively new and emerging technology, with limited knowledge about its effectiveness and limitations. To understand these effects and make the best use of precious public health resources, VOT must be evaluated under more diverse conditions and settings to define its function and compare it with other existing or emerging technologies geared for the same purposes within an evolving landscape (e.g., short message service [texting] communication and electronic medication monitors). Likewise, synergies between digital health and traditional approaches to improve patient treatment outcomes, and even between different digital health technologies, should be explored.

VOT may pose risks to patient confidentiality while data are transferred; however, these issues could be addressed through encryption and secure data management. Any residual risk for disclosure of disease status should be balanced against the likelihood for the same to happen when patients have to visit TB clinics regularly or to have a DOT observer visit their home or workplace every day, which is culturally inappropriate in many societies and could aggravate stigma for the patient’s household.

In early 2015, a multi-partner collaboration, led by WHO and the European Respiratory Society, started to elaborate target product profiles (TPPs) for digital health products focused specifically on topical challenges in the implementation of the new End TB Strategy (*5**,**13*). A TPP describes the characteristics and requirements for a particular concept under development to help different stakeholders, including developers, define solutions to address specific problems. Participants in these discussions define the nature of the problem to be addressed, its relative priority compared with other pressing needs, and match the need to an existing or forthcoming digital solution. Mindful of the positive early results, but also the need for appropriate, evidence-based guidance on its use, the WHO/ERS initiative has identified VOT as one of the digital tools in support of treatment adherence to be followed closely with a TPP (*13*). We propose to take forward the TPP of VOT as a collaborative group of partners. This process will embrace a broad cross-section of representative users, developers, and policy makers. If evidence for the effectiveness of VOT continues to grow, technical details should be elaborated to guide further development and the eventual large-scale deployment of VOT. One of these is the model by which software will be made available, conceivably through open-source or socially responsible licensing (*14*,*15*). Whichever approach is adopted, a sustainable means to enable VOT interventions worldwide, such as through public funds or insurance systems, will be needed.
